# Ethics-testing an eating disorder recovery memoir: a pre-publication experiment

**DOI:** 10.1186/s40337-024-01060-6

**Published:** 2024-08-12

**Authors:** Emily T. Troscianko, Rocío Riestra-Camacho, James Carney

**Affiliations:** 1 University of Oxford, Oxford, UK; 2 University of Oviedo, Oviedo, Spain; 3 London Interdisciplinary School, London, UK

## Abstract

**Background:**

Narratives (including memoirs and novels) about eating disorders (EDs) are typically published with the intention to benefit readers, but survey evidence suggests that reading such narratives with an active ED may more often be harmful than helpful. To reduce the probability of inadvertent harm and learn more about how narrative reading and EDs interact, a pre-publication study was designed to determine whether or not a recovery memoir should be published.

**Methods:**

64 participants with a self-reported ED read either the experimental text (*The Hungry Anorexic* [HA]) or a control text (*Ten Zen Questions* [TZ]) over a roughly two-week period. All participants completed the Eating Disorder Examination Questionnaire (EDE-Q) and the Anorexia Nervosa Stages of Change Questionnaire (ANSOCQ) one week before and two weeks after reading, and answered three recurring open-ended questions at regular timepoints during and after the reading. Computational analysis of the free-text responses assessed text/response similarity and response characteristics on emotional, sensory, and action-effector dimensions. Both rating-scale and free-text data were analysed using mixed ANOVAs to test for effects of time and condition, and the university ethics board was notified in advance of the quantitative threshold for harmful effects that would prohibit the ED memoir from being published.

**Results:**

On the two quantitative measures, there was an effect of time but not of condition: Significant improvement was found in both groups on the EDE-Q (with a medium-to-large effect size) and the ANSOCQ (with a very large effect size). In an ANCOVA analysis, no significant mediating effects were found for age, education, duration of professional support for the ED, or pre/post-reading BMI change. For the free-text responses, linguistic similarity measures indicated that HA responses most closely matched the text of HA, with the same being true for TZ. In a word-norm analysis, text condition significantly affected six emotional, sensory, and action-effector variables (interoception, olfaction, gustatory, mouth, torso, and hand/arm), mean scores for all of which were higher in HA responses than TZ responses. Close reading of readers’ responses explored two potential mechanisms for the positive effects of time but not condition: engagement with the during-reading prompts as part of the experimental setup and engagement with the texts’ dialogical form.

**Conclusions:**

The ED memoir was found not to yield measurably harmful effects for readers with an ED, and will therefore be published. The finding that significant improvement on both quantitative measures was observed irrespective of text condition suggests that positive effects may be attributable to linguistic characteristics shared by the two texts or to elements of the reading and/or reflective processes scaffolded by both. The quantitative results and the free-text testimony have implications for our understanding of bibliotherapy, “triggering”, and the practicalities of responsible publishing.

## Abstract (plain language summary)

This paper reports on an experiment designed to decide whether a memoir about recovery from an eating disorder (ED) should be published. Previous research suggested that reading books about EDs may have strongly negative effects, especially for individuals with an ED. In this experiment, 64 participants were randomly assigned to read either the memoir under investigation, *The Hungry Anorexic* (HA), or a book unrelated to EDs, *Ten Zen Questions* (TZ). Participants completed two questionnaires before and after reading the book over roughly two weeks: the Eating Disorder Examination Questionnaire (EDE-Q), a common measure of eating disorder severity, and the Anorexia Nervosa Stages of Change Questionnaire (ANSOCQ), used to assess attitudes to illness and recovery. Participants also answered three recurring open-ended questions at timepoints during and after their reading. The book would not be published if EDE-Q scores of participants in the HA group worsened significantly, and worsened more than those of participants in the TZ group. Scores on both measures improved significantly between pre- and post-reading for both groups, so the memoir will be published. The positive effects may be due to a feature shared by the two books or a feature of the reading process independent of the books themselves.

## Introduction

### Pro- and anti-recovery effects of narrative reading for eating disorders

We know little about how eating disorder (ED) narratives interact with the trajectories of readers’ illness and recovery. We know rather more about the effects of reading ED-specific self-help books, which are mostly discursive and didactic in form, often with narrative vignettes interspersed for illustrative or inspirational purposes. Reading self-help books with or without guidance seems to generate minor to moderate positive effects [1–4]. The self-help materials are typically treated as a cost-effective substitute for an in-person therapist rather than as texts with linguistic and rhetorical features that may elicit responses usefully understood as “readerly” in nature. As such, the domains of self-help and narrative bibliotherapy have had little dialogue, making it hard to transfer insights between the two.

This study was motivated by the sparse existing evidence regarding helpfulness and harmfulness of narrative reading in the ED context, and by the fact that what evidence there is suggests that harms may be significant when a narrative has an ED focus. The evidence of harmful effects of reading ED narratives also contributed to the lead author’s (ET’s) attempt to write a narrative of illness and recovery designed to maximize the possibility of helping rather than harming readers with an ED. Once the narrative had been written, the experiment was designed to assess the efficacy of the chosen rhetorical strategies by asking whether reading helped or harmed participants, or neither. For the experiment, a harm-reduction focus was adopted: The intention was to reduce the avoidable, measurable harms that might be done by publishing an ED narrative without investigating how readers respond to it.

A harm-reduction angle was adopted because what we do know about responses to ED narratives, fictional or otherwise, is not encouraging. Survey data [5] sampling 885 respondents suggest that reading narratives unrelated to EDs was perceived by a majority of readers with and without a personal ED history to have had positive or neutral effects on the dimensions of mood, self-esteem, feelings about their body, and diet and exercise habits, while reading ED-themed narratives (fictional or nonfictional) was typically perceived as having had strongly negative effects on these four dimensions. A range of potential mechanisms may contribute to harmful effects resulting from ED narrative reading, including exacerbation of ED-relevant behaviours (e.g. by gaining tips from protagonists), an increase in obsessive ED-related thoughts, comparisons between oneself and a character (especially with respect to body or eating/exercise behaviours), and changes in physical sensations or attitudes towards them (e.g. inducing a feeling of fatness or validating a refusal of hunger). In many cases, survey respondents mentioned rapid, powerful, and potentially long-lasting changes, many of them negative and many of them contributing to damaging mind/body/behaviour feedback loops [6]. This evidence directly contradicts theoretical accounts of bibliotherapeutic mechanisms developed in non-ED domains, where what can be characterized as a “similarity thesis” dominates: the notion that narrative reading is more likely to generate therapeutic effects the more closely the protagonist’s situation resembles the reader’s [7].

Beyond the retrospective self-report of Troscianko’s [5] observational survey study and associated analyses [6, 8], a recent experimental study by Riestra-Camacho, Carney, & Troscianko [9] also suggested equivocal effects of reading ED-relevant narratives. The study involved participants reading two full novels in the young adult sports fiction genre, which deals with ED-relevant themes (body, eating, exercise) but without the pathology-focused framing of an ED narrative. In a between-participants design, half of the participants read the novels accompanied by a reading guide designed to emphasise helpful body/eating/exercise-relevant features and interpretations. Those who read the novels with the reading guide showed reduced espousal of gender stereotypes about the body. On other dimensions there were no statistically significant effects in either group, but the data revealed consistent trends towards slight improvement in ED vulnerability when reading with the tailored reading guide and slight worsening when reading without the guide.

Observational and experimental findings of this type do not demonstrate that reading of ED narratives is always harmful, in the context of an active ED or otherwise. Troscianko’s [5] survey study also suggested that reading ED narratives can be helpful, including by giving an idea of what recovery and life after recovery may involve, inspiring with a positive role model, and seeing one’s ED through someone else’s eyes or putting it into perspective. Reading experiences may also contribute to helpful forms of positive (self-reinforcing) mind/body/behaviour feedback or offer sources of stabilizing negative feedback to counter unstable dynamics [6]. Similarly, Riestra-Camacho, Carney, & Troscianko’s [9] finding of a decrease in beliefs about body-related gender stereotypes amongst those using the reading guide suggesting that in the right reading context, ED-relevant (though here not directly ED-centric) material can exert a positive, anti-ED effect.

### Authorial responsibility in eating disorder narratives

#### Publication decisions

In systematic evidence about responses to ED narratives, the potential for harm is currently clearer than the potential for good. There is a striking disconnect between the frequency with which readers report ED-exacerbating effects—sometimes deliberately sought out, often not—and the reasons for writing given by authors of ED fiction or memoir, which typically include the desire to help others dealing with comparable difficulties. As Jones has noted, some authors of memoirs express both their good intentions and the contrasting fact that reading an ED narrative was mostly unhelpful during their own illness: “in some cases, authors claim that their goal is to help or inspire others and include details of how their own reading of ED memoirs and novels fuelled their disordered eating” [10, p315]. Authors’ awareness of the harmful potential of such texts may encourage us to assess their stated aspirations to do good through writing and publication of their own ED narrative as “misguided, or even disingenuous” (ibid.). These considerations support the idea that the writing process and its outcomes for the writer may merit a more systematic distinction from the decision about whether to publish—that is, to make the resulting narrative available to other readers.

There is some precedent in the ED domain for memoir authors making the decision to separate writing from publication. Contributing to recruitment efforts for the current study, recovery coach and blogger Tabitha Farrar created a publicity video [11] in which she describes reaching the conclusion that the ED memoir she had written would be likely to “trigger” many readers and therefore deciding never to publish it. This precedent was discovered only during the recruitment phase for this study, but it indicates that such questioning and decision-making about the pros and cons of publication may be more prevalent than previously understood, in the ED realm or more broadly.

In this study, we required the ED memoir to do no significant measurable harm rather than requiring it to do significant measurable good in order to be published. The high bar of the latter would require an account of bibliotherapeutic benefits far more fully developed than what currently exists. It would also pre-define possible benefits in a way that would circumscribe the wide range of reasons why potential readers might seek out a book. Existing evidence on potential harms, as outlined above, is much clearer, and lends itself readily to assessment with standardized clinical measures as used here. By contrast, what we do know about the therapeutic value of reading suggests that long-term processes of mnemonic and cognitive consolidation are often needed to activate it [12]. Since it would have been impractical to test for these over a timeframe of months or years, we chose to use the weaker but more easily measured “no harm” criterion for publication. In this, we keep company with one of the most fundamental principles of medical practice: *primum non nocere*.

#### Textual construction

In addition to flagging the importance of separating writing from publishing, the existence of data on the anti-recovery effects of reading ED narratives raises other practical questions for authors who do intend to publish, such as how to reduce the likelihood of doing harm through authorial decision-making (a) during the writing process and (b) between writing and publication. Correspondingly, the narrative text being evaluated in the current study was both (a) rhetorically designed to minimize harmful responses, and subsequently also (b) tested with a relevant sample of readers in a controlled manner to guide the decision as to whether or not to publish.

The narrative in question is an autobiographical account of ET’s experience of anorexia nervosa (AN) and recovery from it. Efforts were made to avoid describing an ED in ways that are likely to ridicule, belittle, or glorify the illness. Beyond these efforts to create a responsible account of AN and recovery from it, rhetorical decisions during the writing phase were intended to reduce the likelihood of readers being accidentally or deliberately harmed by reading, via two main factors that differentiate it from many other published ED narratives: (1) a roughly 50/50 chapter split between discussion of the illness experience versus the recovery process and (2) widespread use of dialogue form, including between “interlocutors” who may initially seem to be separate individuals but who turn out to be interacting elements of the protagonist’s own mind.

The evenly balanced proportions of illness and recovery phases differ from the typical ratio found in published ED memoirs, which is heavily skewed towards the illness section of the narrative, often involving “a lengthy description of the extent of the eating disorder and the impact this has on a person’s life” [13, p557]. Although a nominal shift occurred between early ED memoirs (1970–2000) and more recent examples, with the titles in the latter period promising more of a focus on recovery, this is rarely borne out in reality; it typically remains the case that “only a brief glimpse into recovery is portrayed at the end of these books” [14, p89]. In the text under investigation here, a more even balance between illness and recovery is intended to create the opportunity for readers to explore what recovery as a process may involve, as well as what a recovered state may make possible, rather than remaining focused on the illness state or espousing the biomedical terminology of “remission” that presents full and permanent recovery as impossible.

A disproportionate focus on illness in ED narratives may result from a belief that conveying the damage done by an ED will have a therapeutic or preventive effect, but there is little supporting evidence for this belief and increasing evidence to the contrary, as in the research studies cited above. Beyond the ED sphere, research on anti-smoking campaigns has also found “boomerang effects” in which anti-smoking messages can actually increase pro-smoking attitudes and intentions to smoke, potentially because the teenage target audiences perceive smoking as a symbol of rebellion against adult authority, or simply by increasing the salience of smoking via a high volume of anti-smoking material [15]. Both possible mechanisms may have some carryover to the ED context. In more ED-specific terms, evocations of extreme illness may have the unintended consequence of reinforcing the behavioural and value systems of the ED, often by creating a severity comparator that readers may feel they have to live up to. For example, one survey respondent in Troscianko’s [5] study (not quoted in the cited paper) remarked: “I compare myself unfavourably to the ED sufferer in the book. I feel inadequate and worried that I’ve been complacent and not previously realised quite how lazy, fat, etc. i was being and that I need to do more to meet the eating disorder’s required standards because the book just changed the goalposts.” Devoting more significant space than is normally given to the complexities of the recovery process may be of greater value to someone who has no need for “awareness raising” about the damage an ED can do, but who has comparatively little understanding of how recovery might in practice unfold or what it might make possible for them.

The second innovation in the text under investigation, the dialogical format, has some partial precedents in ED fiction and memoir. In *Wintergirls* [16], for example, strikethrough formatting is used to convey alternative perspectives, whether anti-anorexic thoughts or experiences that the narrator does not want to acknowledge or pro-anorexic thoughts or experiences that the narrator gives the impression of semi-censoring for the reader’s benefit. In *Thin* [17], meanwhile, a range of strategies is used to achieve dialogic effects, including sections in the style of a stage play to convey the coexistence of the silent anorexic “voice” and the protagonist’s speaking voice as well as italics to convey unspoken thoughts in sections of narrative prose.

These forms of multiperspectival structure serve in part to convey the ambivalence often inherent to ED experiences thanks to the combination of their egosyntonic qualities [18] with their demonstrable impairment of health and quality of life. As a method for instantiating contradictory perspectives in a sustained manner, the dialogical form employed in most of the book under investigation has a number of potential advantages relative to a more conventional narrative structure with largely consistent internal or external focalization. In particular, it may offer rhetorical benefits by:

1. Allowing the ED logic to be expressed and explored but never to go unchallenged by a perspective of health, common sense, or other contrasting position;
2. Conveying the fact that insight can be high during illness and can also be enhanced through dialogical challenge;
3. Making clear that insight-building dialogue needs to be supplemented by behavioural change if recovery is to begin and be completed;
4. Acknowledging that fully unequivocal commitment and motivation are not necessary for a successful recovery process.

The potential effects of these rhetorical strategies may or may not be observed in real-world reader/text interactions. As Millstein [19, p105] warns, “the assumption seems to be that the message we intend to convey is in fact the message that the adolescent receives. Not only is this likely to be erroneous, it also may be dangerous” (quoted in [15, p424]). How a relevant set of readers would respond was put to the test in the current study, where participants with an active ED read either the ED text in question or a non-ED related control and provided a range of forced-choice and open-ended responses at intervals before, during, and after reading. For the main clinical measure, a predefined criterion was selected to determine the publish / do not publish decision. Specifically, a direction of change and effect size were selected in advance for any potential negative effects reported by participants. The book would not be published if negative effects on the main clinical measure exceeded this threshold. Edits would be undertaken to address any major negative effects reported in the free-text responses, guided by the specifics of these responses.

In practice, all of this meant that we sought to find evidence *against* the null hypothesis that there would be no negative impact of text condition on ED severity, with this negative impact being associated specifically with participants in the *Hungry Anorexic* condition. In the absence of such evidence, publication would proceed. While we were interested on intellectual grounds in other findings, these remained ancillary to our main ambition of testing the effects of HA on its readers.

## Methods and materials

### Recruitment, participants, ethics, and design

This study was conducted between June 2021 and June 2022. It was approved by Oxford University’s Central University Research Ethics Committee. In the study, 64 participants with an ED read either an unpublished ED recovery memoir (*The Hungry Anorexic*, henceforth HA) or a control text unrelated to EDs (*Ten Zen Questions*, henceforth TZ) over a roughly two-week period. They completed two standardized measures before and after reading, as well as responding to a recurring set of open-ended questions during and after the reading.

A sample size of 29 participants per condition was estimated using assumptions derived from prior data published in Troscianko [5], which gave estimates of the effects of ED-themed fiction on readers with EDs. Calculations were performed in accordance with the standard procedures dealing with an equivalence trial with a dichotomous outcome; an alpha of 0.05 and a power of 0.8 were assumed in the procedure. The data in Troscianko [5] were appropriate to use in this calculation because they measured the effects of reading ED-themed fiction compared to non-ED-themed fiction on Diet/exercise habits, amongst other dimensions, for readers with EDs. (Other dimensions tested included Mood, Self-esteem, and Feelings about your body; the behavioural Diet/exercise dimension was chosen as most closely related to the measures being applied in the present study, but other dimensions showed similar effects.) A score of -2 or lower in a scale from -3 to +3 (minor, moderate, or major positive or negative effect) was taken as indicative of a reader experiencing a negative impact of reading an item of fiction. In the ED fiction condition, 52% of responses recorded a negative effect; in the non-ED fiction condition, 3% of responses indicated a negative effect. This provided expected proportions of negative to neutral impacts in each condition. The margin on the risk difference scale was assumed as -0.23 by taking the difference between the mean score in the ED fiction condition and the mean score in the non-ED fiction condition in Troscianko [5], with each being expressed as a percentage of the possible range.

The memoir under investigation, HA, was written by the lead author, ET. The study was designed and carried out in ways intended to minimize the effects of any conflict of interest experimenter bias, including random allocation of participants to the experimental versus the control group and delegation of data curation to RRC and statistical analysis to JC. Ultimately, ET’s motivation to conduct the study derived from her dual status as researcher and writer, and her priority was that the data should robustly guide an ethical decision, whether for or against publication; the study was conceived in an effort to avoid disseminating ED-related material that was likely to do harm, rather than to demonstrate particular benefits.

The study was publicized via university mailing lists, via ED charity and researcher social media accounts, as well as on several blogs and other online channels. To aid recruitment partway through the study, several clinicians were invited to share details of the study with their ED clients if appropriate, and a snowball sampling method was incorporated, by adding an invitation on the last page of the end-of-study survey to share details of the survey with friends or other contacts who might be interested in taking part. Following initially high attrition rates, potential participants were requested not to sign up unless they intended to complete the entire study, and additional suggestions were made to help them plan effectively for the reading portion of the study. In total, 181 individuals were recruited. Of these, 71 completed the initial questionnaires but then did not proceed to the reading phase of the study; only 25 of this subset were recruited after the recruitment changes. The fact that most individuals who dropped out of the study did not even begin the reading, and that retention improved markedly after the changes to the publicity and start-of-study information, suggests that the differences between those who did and did not complete the study concerned primarily time management and possibly also (as touched on in the Limitations subsection of our Discussion) the lower appeal of the non-ED text for some. A total of 65 individuals completed the reading, but the last of these completed the second questionnaire only after the data had been finalized for analysis, so her data were not included, leaving 64 usable submissions.

Potential participants visited a webpage giving more details of the study and providing a link to read the full information sheet and consent form. If the participant chose to submit a completed consent form, her responses were reviewed against inclusion criteria before an invitation to enter the study was provided. The inclusion criteria were as follows: 18 + years old, female, fluent speaker of English, BMI 15+, and self-reporting as being currently in one or more of the following illness/recovery stages: (1) *I currently have a restrictive eating disorder diagnosis*; (2) *I currently consider myself to have a restrictive eating disorder*; (3) *I am currently actively recovering from a restrictive eating disorder*; (4) *I was in recovery from a restrictive eating disorder but my recovery has stalled*; (5) *I was recovering from a restrictive eating disorder but I have since relapsed*. Thus, no participants considered themselves fully recovered; all were somewhere on the continuum from ill and not (yet) attempting recovery to actively recovering. A BMI cutoff was used to reduce the danger of recruiting individuals in a highly compromised mental and physical state, and only females were recruited in order to reduce confounding sources of variance in a sample likely to be predominantly female (see also [9]).

Participants were asked to provide the following additional information: ED type; recovery status (pre- and post-reading); ED duration to date; professional support for the ED/recovery received or not, and if so of what type and for how long (plus any post-reading change in support); age; highest educational level; do you read for pleasure or not; have you ever read a book about EDs, and if so, of what type (self-help, memoir, fiction, or other); BMI (pre- and post-reading). Participants also completed the Author Recognition Test to control for lifetime print exposure or reading volume, which predicts reading skill [20]. On most of these variables, no significant inter-group differences were found. The exceptions were (1) professional support received (22 participants in the HA group versus 12 in the TZ group); (2) support type (HA participants reported more outpatient treatment and counselling); and (3) educational level (but a higher level of undergraduate education in the TZ group counterbalanced the higher level of postgraduate education in the HA group). The groups can therefore be considered broadly well balanced. The majority of participants (55) reported their ED type as AN (including 51 restrictive subtype and 4 binge/purge subtype).

Participants’ free-response data were monitored regularly. Where these gave any indication that the reading experience was having problematic effects, the participant was contacted and invited to share any concerns with ET, either via email or via a video call, and to contact an ED helpline and/or their medical practitioner or therapist (if applicable). We initiated email exchanges of this type with one HA participant (after she noted that she thought the text would be triggering for others) and two TZ participants (one of whom described feeling triggered herself, the other finding the reading experience difficult in other ways). All information shared with ET in this context was treated confidentially and was not included in the data analysis. Participants were also invited to contact ET by email at any point if they wished to raise any questions or concerns about the experiment or their participation. No cause for serious concern was detected, and all three participants who were contacted to check in about their difficulties chose to complete the study.

Prospective/confirmed participants were not informed that ET was the author of the text under investigation until the post-study debriefing was provided, to reduce potential demand characteristics. A few participants indicated having assumed or inferred that ET was the author. A debriefing document outlining the study’s purpose and design was provided when participants were thanked at the end of the study. Three participants who completed the study were selected at random to receive one of three £250 prizes, with payments made via PayPal. Roughly one month after data collection was completed, participants were contacted again with an outline of the main study findings.

The experiment used a between-participants design, in which each participant read the full text of either HA or TZ and data were gathered before, during, and after reading. One week before being provided with text access to start reading, participants completed two validated questionnaires and provided additional information about themselves, their ED, and their reading habits in response to a range of tailored questions. At six evenly spaced points in the experimental or control text (for HA, at the end of each chapter; for TZ, at the end of each of six sets of several shorter chapters) participants answered three recurring open-ended questions (discussed below). Participants were requested to aim to complete their reading within a two-week period, and were offered guidance on how to plan for this. Then, approximately two weeks after completing the reading, participants repeated the two validated questionnaires and a subset of the other demographic questions, plus an adapted variant on the three open-ended questions. Participants were asked not to read any other books about EDs during the five weeks of the study. This request did not exclude textual materials encountered in any other formats (blogs, online articles, etc.), but it was intended to reduce potential confounding effects from engagement with other book-length ED-themed texts.

### Text choice and presentation

The control text was *Ten Zen Questions* (2009) [21], reissued as *Zen and the Art of Consciousness* (2011), by Susan Blackmore. The author granted permission for the full text of her book to be used for the study. Both texts are meditative in nature, centred on in-depth exploration of psychological questions including the nature of self and identity, and both have a strong dialogic element; this means that they are likely to entrain similar cognitive states, although only one by activating ED-related cognitions. For instance, in HA the dialogue is made explicit through labelling of multiple aspects of the self as separate interlocutors, often in question-and-answer form. The first chapter is called “The basics”, and its first section, “Catching anorexia emerging”, begins:

- B: So, what was it like, heading off to university with the all-clear from the child psychiatrist?
- A: Um, pretty bleak.
- B: That’s it? Bleak?
  …
- B: OK, what kind of bleak?
- A: Ugh, the room, the horrible little room they gave me. As soon as I walked in the smell of depression hit me like nausea. And when my parents left and I went off to buy milk and then I was back there again, waiting for the kettle to boil—god, it was almost over before it began.
- B: Couldn’t you just have *tried* a bit?

In TZ the dialogue is instantiated via a question-and-answer form at the level of the book’s macrostructures (each chapter is devoted to a separate question, in the form of a Zen koan) as well as its microstructures (accumulating repetitions-with-variations on the questions and answers). For example, the first chapter is entitled “Am I conscious now?”. It begins:

- Of course I am. Yes, I am conscious now.
- Am I conscious now?
- Of course I am. Yes, I am conscious now.
- But something odd happened. When I asked myself the question it was as though I became conscious at that moment. Was I not conscious before? It felt as though I was waking up—coming to consciousness when I asked the question—because I asked the question.
- What is going on? (Calm down. Take it slowly.) Am I conscious now?
- (p. 41)

TZ is significantly shorter than HA (50,164 versus 132,665 words). The texts were presented in matched formatting (font type and size, margin size, etc.).

Following the procedural precedent established by Riestra-Camacho, Carney, & Troscianko [9], the texts were presented as PDFs within Google Drive, which allowed the files to be shared with a limited number of people (the participants), giving them permission only to view the file and preventing them from copying, editing, downloading, and sharing it with others. Participants could thus read the text on their device of choice, in their own time and in their preferred reading environment. Each participant had access to their own file and when each finished reading, their access was removed. The recurring questions were presented at the end of the relevant chapter/section in the form of user-generated comments (tagged as posted by “Experiment team”) in the right-hand margin, using the sticky-note function. Participants responded to the questions directly within each sticky. Four participants had difficulty reading the Google Drive version and requested a downloadable copy of the text to annotate on their own device and email back to the researchers.

### Clinical questionnaire measures and free-text response questions

The primary clinical measure, presented pre- and post-reading, was the Eating Disorder Examination Questionnaire (EDE-Q) [22], a widely used measure of ED severity with acceptable internal consistency and fairly strong test–retest reliability [23]. The EDE-Q was chosen to serve as an indicator of changes in the core ED pathology (both psychological and behavioural) that might occur during the reading period or in the two weeks afterwards. The book would be considered unusably dangerous (beyond the point where edits could help) if (1) deterioration on the EDE-Q scoring was observed, (2) deterioration was greater in the experimental group, and (3) the effect size of the difference between conditions was large, as defined by Cohen’s d (> 0.8). The Anorexia Nervosa Stages of Change Questionnaire (ANSOCQ) [24], which has good construct and predictive validity and internal consistency [25], was administered as an additional measure, designed to tap the individual’s attitude to her illness and recovery on a continuum from pre-contemplation through contemplation, preparation, action, and maintenance. If the act of reading did not change the current state of the core cognitive-behavioural ED markers, it might nonetheless exert effects on attitudinal relations to the ED, which may be more malleable in the short term. Change on the ANSOCQ scale was not considered adequate grounds for preventing publication entirely.

The full-text PDFs contained stickies posing three recurring questions six times, at the end of a chapter or set of chapters, as follows: *How is your day going? How do you feel about your illness and/or recovery right now? How have you found the experience of reading this section of the book?/[at timepoint 7, in online survey] How did you find the experience of reading the book as a whole?* These questions were intended to generate open-ended responses about the reading experience as well as about the wider context, both the day and life unfolding around the reading and also the attitudes to illness/recovery that might be shifting in response to the reading and/or for other reasons.

Although the decision as to whether or not to publish was made with respect to validated clinical measures, there is an independent value in assessing free-text reader responses. These allow the impact of the text to be assessed on the reader’s own terms, which can yield important data not captured by the numerical scoring of responses on a scale. This is especially relevant when it comes to evaluating the impact of textual exposure, which by definition is linguistically mediated.

Following the precedent established in Troscianko & Carney [26], we combined quantitative and qualitative methods by using word-norm data to numerically score the free-text responses provided by readers, enabling us to conduct statistically robust comparisons between and within readers and sidestep the problem that purely qualitative analysis is significantly informed by pre-existing experimenter expectations. Word-norm data consist of large corpora of words that have been rated for their impact on various cognitive and affective dimensions (for example, the extent to which a word is experienced as abstract or concrete, or pleasant or unpleasant). They are a stable numerical measure of how much a word is associated with a particular quality as measured across multiple participants [27]. Since the impact of any given word has been established with respect to enough participants to establish a high interrater reliability (usually on the order of 20 or so raters), word norms provide a useful metric for the population-level reception of the word. In this study, we used word-norm data related to emotional and somatosensory impacts of words, as both dimensions of impact are relevant to ED- and recovery-related cognition.

**Fig. 1:**
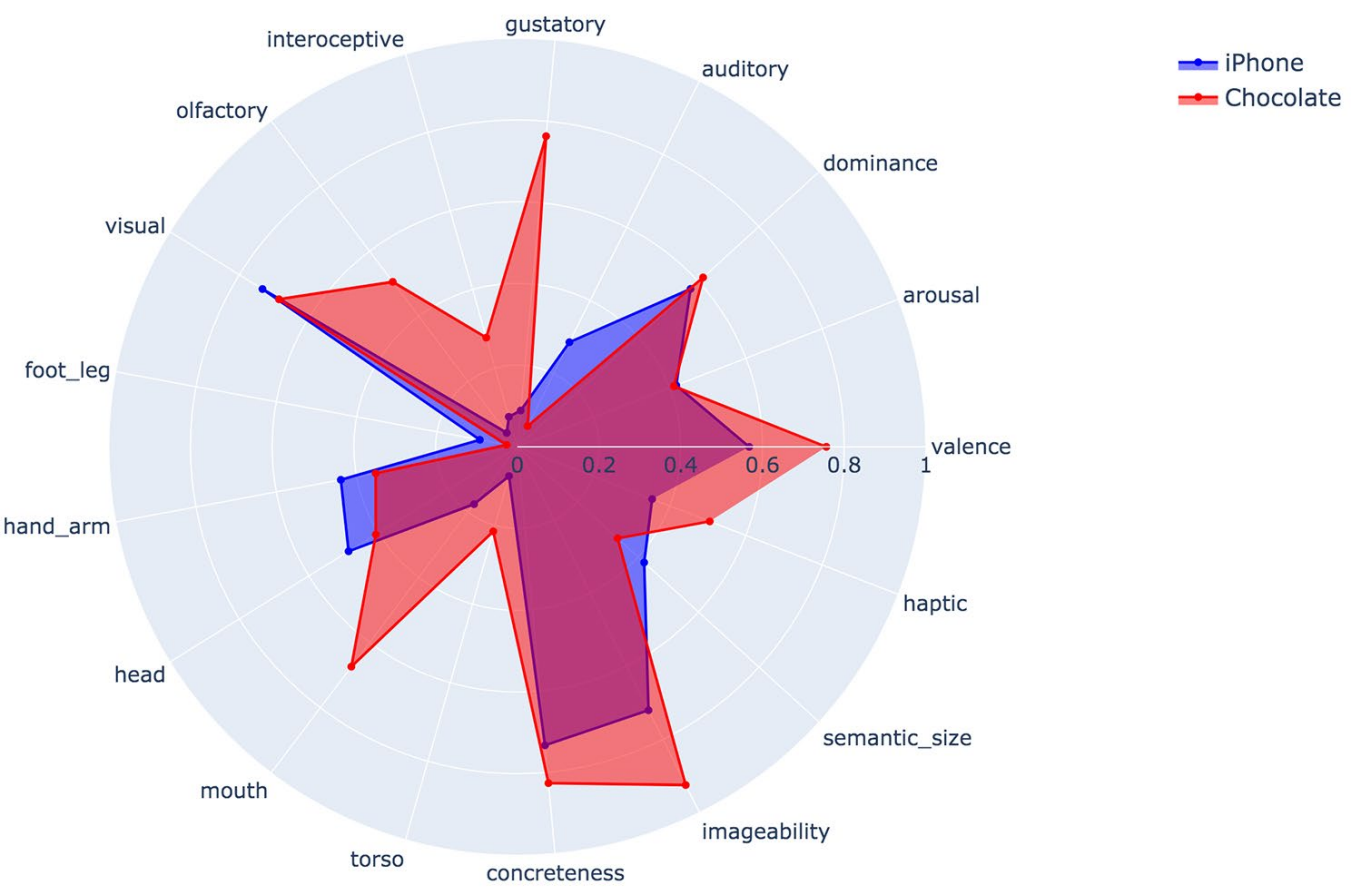
Word norm ratings of the words **chocolate** and **iPhone**. The numerical value associated with each category is the average of consistent responses across raters, with consistency being measured using a reliability measure like Cronbach’s alpha

The emotional associations in the free-text data were captured using the norms for valence, arousal, and dominance (VAD) published in Warriner, Kuperman, & Brysbaert [28]. Following Mehrabian & Russell [29], this dimensional approach to emotion suggests that any emotional stimulus can be decomposed into how positive or negative it is (valence), how energizing or sedating it is (arousal), and how in-control or controlled it makes a person feel (dominance). Thus, a word like HOLIDAY is high in valence, a word like DANCE is high in AROUSAL, and a word like DEMENTIA is low in dominance. The somatosensory freighting of response data was calculated using the Lancaster sensorimotor norms [30]. These norms deliver scores of how much a word is associated with a perceptual modality or motor effector of the body. There are 11 of these in total, corresponding to the five senses plus interoception (the experience of sensations inside the body), and five motor effectors (hand/arm, foot/leg, head, mouth, and torso). One insight yielded by the Lancaster norms is that even highly abstract concepts like JUSTICE and ESSENCE have a consistent sensorimotor coding. Concreteness and imageability of words used were measured using the ratings collected in [31], where concreteness captures sensory salience of the word and imageability the ease with which a mental image of the word’s referent can be generated. See Fig. 1 for word-norm ratings on two common words.

## Results

### Clinical questionnaire responses

**Fig. 2:**
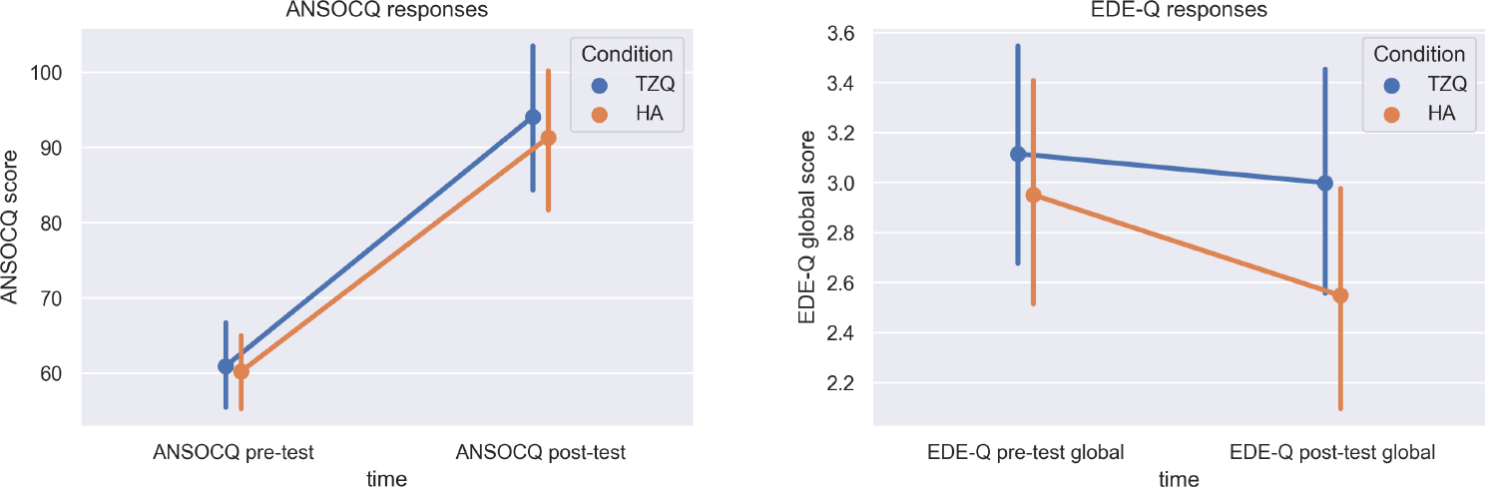
Results for ANSOCQ and EDE-Q questionnaires

Responses to the EDE-Q and ANSOCQ questionnaires were analysed using a mixed ANOVA design, where time of administration (before reading, after reading) was the within-participants factor and text read (HA, TZ) was the between-participants factor. For the EDE-Q, the global response measure was taken as the dependent variable; for the ANSOCQ, the dependent variable was the total score. EDE-Q responses showed no effect of text exposure, but time of administration showed significantly lower scores in the after-reading condition (*DF* = (1, 62), *F* = 8.69, *p* = .004). The η2 effect size of 0.12 indicates that this is a medium-to-large effect (Fig. 2). Results for the ANSOCQ followed a similar pattern, with there being no effect for text exposure but a statistically significant effect for time (*DF* = (1, 62), *F* = 124.83, *p* < .001). The η2 effect size of 0.66 indicates that this is a very large effect (Fig. 2). Analyses were conducted using the pingouin statistics library for python [32].

The key outcomes are therefore that (1) there is no statistically significant effect of text read and (2) there is a substantial and statistically significant effect of time of test administration. Clearly, these results do not challenge the null hypothesis that there is no effect of text read on performance in either the EDE-Q or the ANSOCQ. Nevertheless, they are consistent with the view that reading has a positive impact on the variables measured by these questionnaires, allowing that other non-measured variables may also be responsible for the effect of time.

A separate ANCOVA test was performed to establish whether these results were due to the effect of covariates in the form of age, education, duration of professional support for the ED, or pre/post-reading BMI change. Controlling for the effects of these variables had no impact on the model so they could be discounted as mediators of the changes observed.

With a view to establishing whether recovery stage category influenced responses on either the EDE-Q or the ANSOCQ measure, four two-way ANOVAs were performed. That is, for the each of the EDE-Q and ANSOCQ measurement scales, change scores (the difference between pre- and post-test scores) were taken as a dependent variable, and the change score means for each of the recovery stages were compared. The between-participants factors were text condition and recovery status. Since there were small differences between recovery status recorded in pre- and post-test conditions (some individuals changed their recovery status), this was done for both pre- and post-test recovery stage data. It should be noted that recovery stages were highly imbalanced: There was only one observation in each group for category 2 (“I currently consider myself to have a restrictive eating disorder”) in the post-reading recovery status reports; and there were no observations of category 5 (“I was recovering from a restrictive eating disorder but have since relapsed”) in the TZ group and only two in the HA group in the post-reading reports. At the pre-reading recovery status observation point, there was only one instance of category 2 in the HA condition and no observations in the TZ condition. Across all combinations of experimental conditions and reading recovery status, only category 3 (“I am currently actively recovering from a restrictive eating disorder”) had substantial presence. This meant that recovery stages were not distributed evenly between experimental conditions, so statistical inferences could not reliably be made. Once the two categories with too few observations for any kind of inference at all were removed (as they had counts of 0 or 1 so had no variance), no statistically significant differences between means were found for recovery category in the EDE-Q or the ANSOCQ, whether in the pre-test or post-test category. This suggested that recovery status had no impact on results, allowing that the inferences were underpowered in some recovery categories.

### Computational analysis of free-text responses: emotion, sensory, and action-effector dimensions

Free-text responses consisted of replies by participants to a fixed set of three questions at six staged timepoints. (For this analysis, we excluded the separate end-of-study free-text responses, in which participants reflected once more on their day and their illness/recovery-related feelings and commented on the experience of reading the book as a whole and on taking part in the study.) Responses varied widely in length, from only a few words per answer to a maximum of over 1400 words across the three questions for one HA participant at timepoint 1. These data were lemmatized using the spaCy natural language processing library, which reduced redundant lexical variation by mapping each word onto its root form (i.e. “ran” and “running” would both map to “run”.) Responses were then evaluated for emotional, somatosensory, and action-effector associations using word-norm ratings as described in the preceding section.

Data were analysed using a mixed ANOVA, where time was the within-participants factor and text condition was the between-participants factor. The dependent variable was the score of participants’ responses on a particular linguistic variable. Eight variables showed significant differences on the between-participants factor (text condition): gustatory, haptic, interoceptive, olfactory, foot_leg, hand_arm, mouth, and torso (Table 1). In all cases, mean scores were lower in the TZ condition than in the HA condition, pointing to a common factor potentially underwriting these responses.

**Table 1:** Between-group differences for linguistic variables

Variable	DF1	DF2	F	*p*	η2
Gustatory	1	59	7.10194821	< .001	0.10743932
Haptic	1	59	9.18323714	.00362271	0.13468468
Interoceptive	1	59	5.10555835	.02755666	0.07964299
Olfactory	1	59	8.96658589	< .001	0.13192638
Foot_leg	1	59	23.2960441	< .001	0.28307611
Hand_arm	1	59	10.0475157	.00241893	0.14551596
Mouth	1	59	26.8776334	< .001	0.31297594
Torso	1	59	33.440592	< .001	0.36175225

DF1 denotes the degrees of freedom in the experimental condition; DF2 by participant. The test statistic, F, compares the variability between group means to the variability within the groups. The significance, threshold, *p*, denotes the likelihood of these differences occurring by random chance. The effect size, eta squared, ranges from 0 to 1, where 0 indicates that the factor explains none of the difference between group means and 1 indicates that it explains all of it

**Fig. 3:**
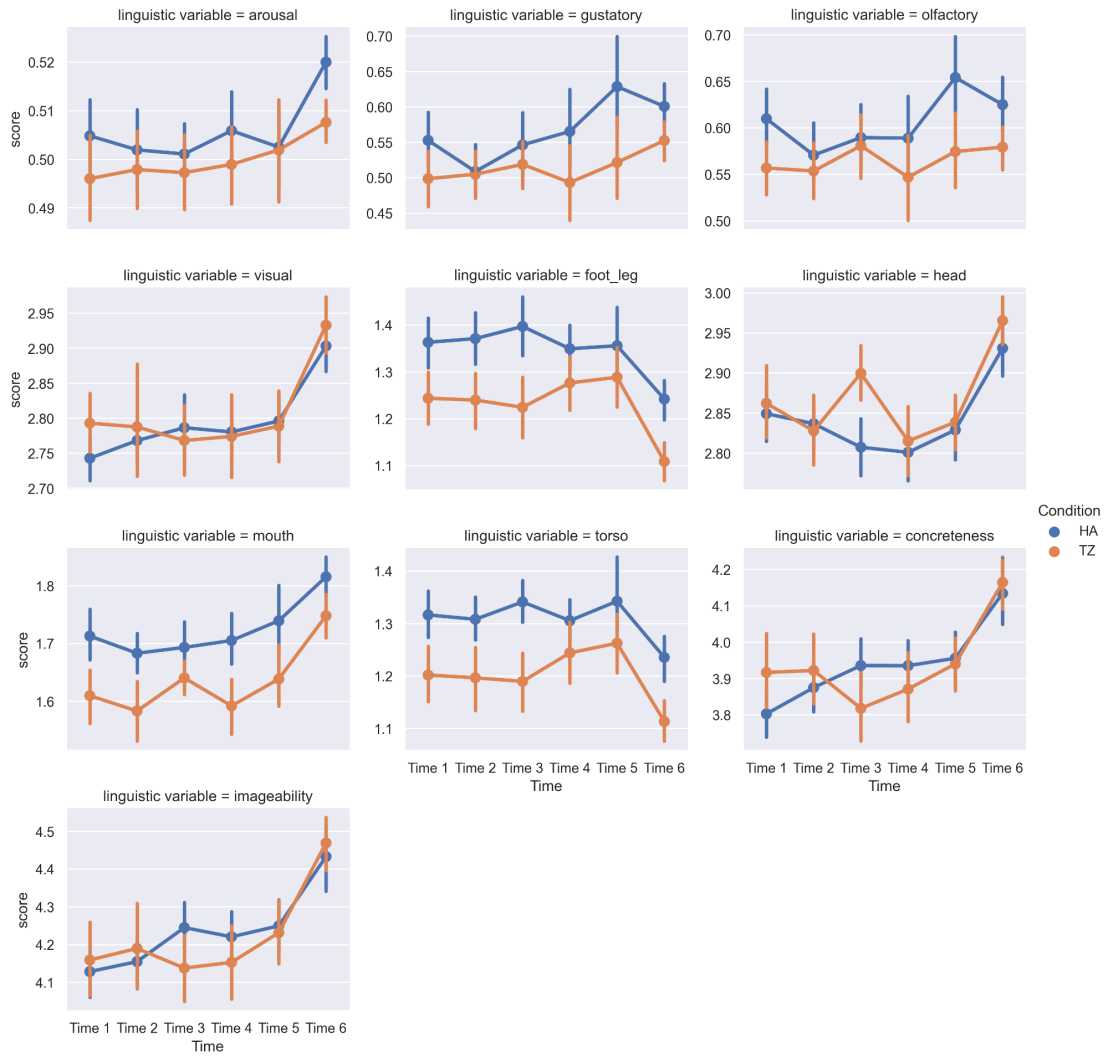
Linguistic variables over time. All variables are scaled between 0 and 1, where 0 is the lowest possible score and 1 is the highest

With respect to the within-participants factor (time), statistically significant results were considered only if there was a consistent pattern of increase or decrease across timepoints (Fig. 3). In this regard, the relevant results were for arousal, gustatory, olfactory, visual, foot/leg, head, mouth, torso, concreteness, and imageability (Table 2). A separate ANCOVA analysis showed no significant effects of age, duration of professional support, education, or BMI change when these were included as covariates in the model.

**Table 2:** Within-participant differences for linguistic variables. See Table 1 for further details of notation

variable	DF1	DF2	F	*p*	η2
Arousal	5	295	5.54798848	*p* < .001	0.08595138
Gustatory	5	295	4.66796417	*p* < .001	0.07331731
Olfactory	5	295	3.50287512	*p* < .001	0.05604342
Visual	5	295	17.2277886	*p* < .001	0.22600405
Foot_leg	5	295	8.62660279	*p* < .001	0.12756227
Head	5	295	14.5630357	*p* < .001	0.19796676
Mouth	5	295	10.6764492	*p* < .001	0.15322895
Torso	5	295	5.49597271	*p* < .001	0.0852142
Concreteness	5	295	14.4453418	*p* < .001	0.19668152
Imageability	5	295	16.7530409	*p* < .001	0.22115338

### Text/response similarity

**Fig. 4:**
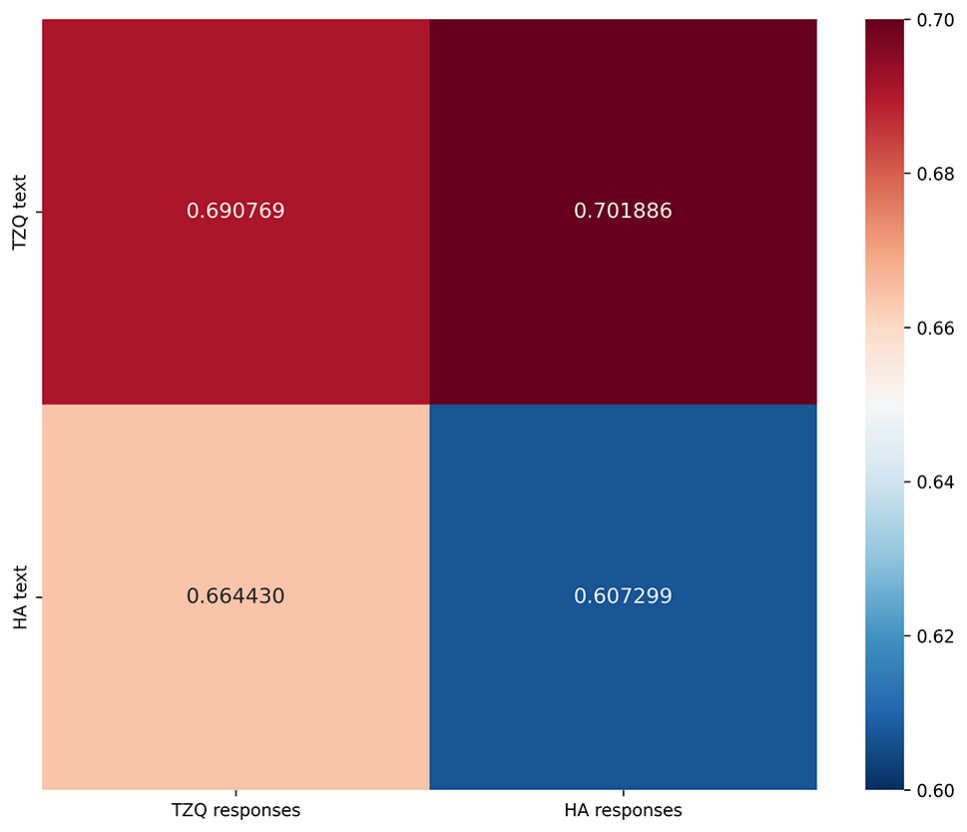
Jensen-Shannon divergences between texts and responses

While word norms are useful for capturing differences in response patterns with respect to emotional and somatosensory language, there is also a value in investigating whether the specific words used in each text influenced the words used in the free-text responses. We did this using an information-theoretic measure called the Jensen-Shannon divergence (JSD), which captures how closely two probability distributions match each other when they do not contain exactly the same items. (In this, it differs from the more commonly used Kullback-Leibler divergence, which requires the same number of items in both distributions.) We calculated the JSD with respect to the word frequencies of the two texts and the corpus of words used by participants in each experimental condition. As expected, the TZ text was closer to the TZ responses relative to the HA responses, just as the HA text was closer to the HA responses relative to the TZ responses (Fig. 4). This is consistent with each text playing an organizing role in shaping the language used by participants when responding to it. The divergence was lower for HA, indicating that it possibly exerted a greater influence than TZ.

### Close reading of free-text responses: engagement with dialogue form and experimental setup

The main experimental results for the EDE-Q and ANSOCQ present something of a puzzle. While there is no significant effect of text read, there is a large effect of time for both texts. How might we explain this? In this section, we look at the common factors that might explain the shared effect of time. Two obvious candidates are that time is mediating (1) positive effects of responses to the question-and-answer and other dialogical structures shared by both texts and/or (2) positive effects from textual engagement as structured by the experimental setup. We tested these possibilities by qualitatively coding and collating participant responses as they bear on either of the two. We acknowledge there may be other implicit variables at work that do not feature in our analysis, but as we cannot know what these are, we chose to proceed with the most plausible explanations.

In Tables 1 and 2, indications are given of how many responses aligned with a specific theme or subtheme and how many individual participants these responses were provided by. As with any qualitative analysis, however, the categorizations are subjective and should be treated as indicative of broad frequency trends rather than highly precise comparisons. The participant quotes from which the themes and subthemes were derived can be found in the supplementary materials.

#### Responses to dialogue and question/answer structures in both texts

One obvious way to account for the improvement on time without a difference by condition is that elements of the texts themselves, but elements distinct from the central thematic emphasis, are responsible for the positive effects. The stylistic feature that most clearly links the two texts is the dialogic, questioning nature of the textual construction. Many participants in the HA group mentioned having found the dialogue form (or the “conversation with [an] anorectic mind”, as one participant put it) compelling, some reflecting on its effects at length. The dialogue form also elicited negative reactions for a similar number of HA participants, the majority of which involved confusion about the identities of the interlocutors. Comparable testimony to both the engaging and the challenging, confusing, or offputting effects of the formal construction were also found in many TZ participants’ responses. One remarked at timepoint 4, for instance, that “I found the discussion of reflective and answerless questions quite calming - odd, since usually not knowing the answers makes me anxious”. Table 3 shows the responses that touched on the effects of the dialogical or interrogative structures of the texts.

**Table 3:** Readers’ responses mentioning dialogue or question/answer form

Identifier	Comment quantity	Themes
Response set 1: Positive responses to dialogue form (HA)	13 (13 ppts)	● Finding internal dialogue relatable ● Curiosity about interlocutors’ identities ● Compelled, interested, engaged, or thoughtful response to dialogue form ● Finding style easy to read ● Answering the questions for oneself while reading ● Perceived similarity between own experience of AN and dialogue form of text ● Usefulness of “voice of reason” role in text as counterpoint to AN voice ● Reassurance found in text’s gradual shift from dialogue to monologue (as protagonist’s self grows more unified) ● Experiencing reading as therapeutic or cathartic
Response set 2: Negative responses to dialogue form (HA)	18 (14 ppts)	● Confusion about interlocutors’ identities ● Criticism of perceived dissimilarity with own ED experience ● Experience of dialogue form as frustrating, annoying, unenjoyable, impersonal, disjointed, over-long, self-indulgent, faux-poetic, or surreal
Response set 3: Neutral/mixed responses to dialogue form (HA)	7 (7 ppts)	● Experience of dialogue form as surreal or unsettling ● Experience of dialogue form as confusing but enjoyable, or as overwhelming but hopeful ● Ambivalence about perceived similarity with own ED experience ● Neutral observation of perceived dissimilarity with own ED experience ● Mixed feelings about similarity and comparisons of self with protagonist voices
Response set 4: Positive responses to dialogue form (TZ)	5 (4 ppts)	● Enjoyment of open-ended questioning ● Appreciation of intellectual challenge and meta-perspective on human thought processes ● Extension of mindful question-asking into everyday life beyond the reading ● A feeling of companionship in question-asking
Response set 5: Negative responses to dialogue form (TZ)	6 (6 ppts)	● Lack of interest or engagement ● High cognitive load: tiring, hard to concentrate on ● Confusion, frustration, annoyance ● Interpreting style as circular, pointless, self-indulgent, or indicative of mental illness ● Need to reread sections
Response set 6: Neutral/mixed responses to dialogue form (TZ)	12 (12 ppts)	● Intrigue or appreciation mixed with impatience or sense of pointlessness ● Difficulty, mental workout, need to reread to check comprehension ● Enjoyment of questions but desire for clearer answers ● Appreciation of mind-expanding effects but with sme frustration/tedium ● Observation of parallels with anorexic thought patterns

#### Responses to experimental setup

It also seems plausible that the nature of the regular answering of open questions at intervals during the reading may have affected the participants’ interpretive processing. Substantiating this possibility, a number of participants in both groups indicated awareness of the researchers as recipients of their responses, including via chatty asides or inclusion of thanks, apologies, and other social niceties. More substantially, some individuals also mentioned other positive and negative responses to the recurring questions or appeared to use them for autoregulation or self-coaching purposes. One TZ participant wrote at timepoint 5, for example: “Frustrated that I am still at the same stage in my recovery as I was 6 months ago. I am bored of being unwell and I want my life to move on. I am angry that the ED is still affecting my life. I need to find other coping mechanisms.” Table 4 summarises the participant free-text responses most relevant to understanding their engagement with the experimental prompts.

**Table 4:** Readers’ responses to experimental prompts during reading

Identifier	Comment quantity	Themes
Response set 7: Positive reactions to recurring questions or questionnaires (HA and TZ)	8 (8 ppts)	● Appreciation of consistency of recurring questions as prompts to reflection ● Appreciation of questions as alternative to speaking with someone else about ED ● Assessments of questions or question-answering as interesting, enjoyable, relevant, useful, or insight-generating ● Using questions as a way to summarise thoughts and feelings ● Awareness of researchers as recipients of responses
Response set 8: Negative reactions to recurring questions or questionnaires and suggestions for improvement (HA; no examples observed in TZ)	5 (5 ppts)	● Experience of questions as too frequent or too broad ● Difficulty answering the questionnaires ● Preference for opportunity to comment anywhere in the text, talk to someone directly, or record voicenotes ● Wish to be able to ask author questions about book
Response set 3: Question-answering as autoregulation or self-coaching (HA and TZ)	8 (7 ppts)	● Honest reflection on current eating/exercise habits, ideas or self-motivation for altering them ● Self-talk to articulate and challenge ED thoughts or emotions, move through dissonance or difficulty ● Reinforcement of need for change ● Active articulation of gratitude or acceptance

Here we have posited two broad speculations as to possible contributors to the interesting main effects found in this study. In both domains, a mixture of responses that can be interpreted as positive, negative, mixed, or neutral may have contributed to the overall positive effects observed on the two standardized measures, since complex dynamics of enjoyment and unease, engagement and difficulty, can unfold in a way that is nonetheless globally beneficial. As one TZ participant put it, “Lots of up and down’s while reading the book. Her voices where so similar as some of mine’s. Mixed emotins, but at the end I think it has been a good experience.” In the Discussion, we explore further the potential mechanisms and implications of the text-prompted changes observed in this study.

## Discussion

In this study, 64 participants with a self-reported active ED read either an unpublished ED recovery memoir or a control text unrelated to EDs over a roughly two-week period and completed the EDE-Q and ANSOCQ one week before and two weeks after reading, as well as responding to three repeating open-ended questions at regular timepoints during and after reading. The intention was to establish whether any change in ED severity was manifested after versus before reading in order to guide a yes/no decision as to whether to publish the memoir. We also sought to investigate the phenomenology of the reading process as it relates to the processes of illness and recovery and individuals’ attitudes to them, to enhance understanding of these response dynamics as well as to guide pre-publication edits to the recovery memoir.

### Clinical measures

In this pre-publication study of readers’ responses to an ED-themed memoir and ED-unrelated control text, both ED symptomatology (as measured by the EDE-Q) and ED attitudes (as measured by the AN-specific Stages of Change Questionnaire) were improved for post-reading versus pre-reading for both the experimental (HA) group and the control (TZ) group. Both groups manifested significant positive change with effect sizes that were moderate-to-large (for the EDE-Q) or very large (for the ANSOCQ).

These findings run counter to widespread intuitions (for example amongst many clinicians) that in general reading may have minimal effects on ED-relevant factors, perhaps especially core symptomatology. They also contradict existing evidence suggesting that engagement with texts may have modest or no detectable positive effects on ED-relevant dimensions, including Riestra-Camacho, Carney, & Troscianko’s [9] study of young-adult sports fiction and much research in self-help bibliotherapy. In self-help bibliotherapy studies, even self-help books designed for the sole purpose of eliciting therapeutically valuable responses typically generate only minor to moderate positive effects, with research remaining inconclusive about many basic questions of efficacy and mechanisms. The results also diverge from the strong retrospective self-report evidence in Troscianko’s [5] survey study that ED-specific narratives can have rapid and significant harmful effects on core ED dimensions. Finally, the results also challenge the “similarity thesis” (the expectation that therapeutic ED-related effects depend on thematic congruity between the textual content and the reader’s health situation) since here an effect was found only of time and not of condition—that is, engagement with the ED-themed and the ED-unrelated text had equally positive effects.

The study’s main findings are compatible with a popular belief that engagement with narrative or “literary” texts exerts strong effects on readers—effects typically assumed to be positive in nature. This belief underpins narrative bibliotherapy, which seems to be a relatively common informal practice in one-to-one therapeutic settings, and it is implicit in much humanities research and many public-facing literary arts initiatives. However, the results do not unproblematically support the thesis that “books do good”, because it is unclear which features of these two texts may be primarily responsible for the observed improvements, or indeed to what extent the textual features themselves were a major driver at all.

Beyond the impact of text, the absence of mediating effects found in the ANCOVA for either the EDE-Q or the ANSOCQ of any of the main continuous variables (age, professional support duration, highest educational level, BMI change) suggests that the main effects are not significantly susceptible to contributions from demographic or ED severity/support variables, nor indeed from the text choice itself. This null finding further supports the idea that the benefits derive from facets of the text/reader interaction that have yet to be confirmed.

In the remainder of this section, therefore, the free-text response data will be used to generate provisional conclusions as to the mediators of the two main effects and related phenomena. We begin with the computational analysis on three analytical dimensions, offering suggestions as to the drivers of effects related to emotional variance, somatosensory effects, and text/response similarity. We conclude by offering insights generated by close reading of participants’ reactions to dialogue form and the experimental setup. Unedited illustrative quotations from participants’ responses are included as subsection epigraphs and as part of the main discussion. Unless otherwise indicated by bracketed ellipses ([…]), all quotes represent the entirety of a participant’s response to a given question.

### Computational analysis of free-text responses

#### VAD dimensions

“I started this push at recovery a year ago, and feel it has failed. I can’t believe how hopeful I felt back then. I feel so ashamed at how I’ve behaved in the interim; it seem perverse that recovery should have destroyed my relationships more effectively than anorexia. I don’t think I nor my family had expected quite how much anger and fear and emotionally-charged reforging of me would need to be done - we’ve all been shocked. Pity is easier to give, if not to receive. What do you give someone who is explosively recovering?” (HA participant)

Using the VAD norms to analyse participants’ free responses (Fig. 3) found a significant within-participants effect for arousal, which increased across the six reading timepoints, and no effects for valence or dominance. The increase in arousal was especially marked for timepoint 6 in HA responses, where an unexpected death and mourning process are described. This thematic shift intensifies the general expansion out from ED-centric themes and experiences that characterizes the later stages of ED recovery, as noted by one HA participant: “Different than the previous sections. More about other life challenges than food-specific ones, which would reflect life after recovery.” Three HA participants at timepoint 6 used language that is particularly rich in high-arousal words, including “rollercoaster”, “climax”, and “inspired and scared”. Arousal increased more steadily across TZ responses between timepoints 3 and 6, and there were no striking examples of high-arousal language in the TZ free-text responses.

Arousal and dominance are typically inversely correlated, but in responses to both texts, the dominance as well as the arousal peak was observed at timepoint 6, suggesting that the narrative progression in both texts—and/or the processes involved in responding to them—had both arousing and control-enhancing elements. The absence of significant change over time in valence values in both groups, and free-response mentions of enjoyment or otherwise that demonstrated no obvious pattern either, suggest that liking and dislike did not play a major role in any reading-related effects. This aligns with findings from a group-reading setting, in which liking or disliking the text being read did not correlate with the perceived value of participation, and liking or disliking the group discussion did not usefully capture the emotional variation in these sessions [33]. One participant whom we contacted because she seemed to be finding the reading emotionally difficult clarified that although she was not enjoying the book very much and it would not be her preference stylistically, the reading and question-answering was encouraging meta-reflection on her thought processes and greater honesty about her current situation. The VAD results and comments of this kind further encourage an interpretation of the main findings according to which the processes of engagement trump the textual features in driving effects in readers—and adds the further hypothesis that enjoyment of these processes is not a major predictor of their value.

#### Sensory and action-effector dimensions

“Confident and exhausted at the same time. Exhausted because everyday I ‘get something wrong’: I don’t manage to stop bingeing, I can’t force myself to eat afterwards, or because I’m afraid to challenge myself. Confident becase sometimes, when I give up controlling and micromanaging, wonderful things happen, giving me hope. Yesterday I had a proper and elaborated afternoon snack (not only an apple, but yogurt with rice cakes and peanut butter, and fruit) and unexpectedly I stopped eating when I felt ok. No binge eating on peanut butter as usual.” (HA participant)

As for the sensory and action-effector dimensions, the increase in gustatory values between timepoints 2 and 5 in the experimental group is explicable with reference to the increasing textual emphasis on food and eating. HA naturally contains a much higher density of food-related terminology than TZ. A number of HA participants mentioned being bored, overwhelmed, or otherwise put off by the amount of food-related language in the middle and later sections of the book; these reactions may partially account for the increase in gustatory language for this group.

Olfactory and mouth norms tend to track gustatory ones; concreteness and imageability, which tend to co-vary, may also be driven by the gustatory language. The marked increase on visual ratings at timepoint 6 in both groups suggests a possible effect of the use of visual metaphors for increased cognitive “insight” or a more “zoomed-out” perspective at the end of the two books, corresponding to textual elements in both that convey a trajectory of culmination. The similar pattern for “head” may also be tied to the use of visual metaphors for cognition. The decreases in foot/leg and torso at timepoint 6 are not readily accounted for, but may be inverse correlations with an increase in cognitive insight. Body-related language mostly concerned difficulties with body image and bodily sensations, worries about fat, and other negative body-related feelings and attitudes common in ED experience.

#### Text/response similarity

“Interesting. It’s a challenging read and different to what I’d usually go for. But I’m getting a lot from it, and find the writer’s voice engaging and very clear. As she’s struggling to come to grips with some of these very confusing questions, she’s bringing us, the readers, with her on her journey. Am I conscious now? And now? It really is like waking up as soon as the question is asked. Very interesting!” (TZ participant)

Analysis of similarity between the texts and responses to them found that the responses for TZ most closely matched the text of TZ, and the same was true of HA, indicating that both texts significantly shaped the language choices of the participants answering questions subsequent to reading them. Without a linguistic corpus to provide a baseline for divergence, relative strengths of these effect for HA versus TZ are hard to assess, but a possibly stronger effect for HA may reflect a greater thematic capture of ED-related material, as suggested by the higher frequency in the TZ group of comments to the effect that they had found the text boring to read or struggled to concentrate on it. Specifically, 15 HA vs. 23 TZ participants mentioned being bored or not engaged or put off at one or more points in the text. These responses may reflect the difficulty of concentrating on non-ED-related stimuli when cognition is impaired by an ED; and this kind of “cognitive constriction” [5, p12] may be especially marked when an ED is associated with malnutrition [34]. As one might expect, valuing cognitive/emotional exploration showed the inverse pattern from boredom/disengagement across the two groups, with more comments to this effect in HA than TZ (20 HA versus 14 TZ). Overall, close reading suggests that TZ was somewhat more polarizing than HA in terms of liking or enjoyment, perhaps in part because of the lower cognitive-emotional capture of non-ED material. These differences, combined with the overall trend towards lower values for TZ than HA on the sensory and action-effector dimensions, suggest that ED-themed material may have a more directive channelling effect on cognition, offering some support for the cautionary angle predicated on the intuition that these texts in some sense “land harder”.

### Close reading of free-text responses: textual and contextual factors

Beyond the three types of computational analysis, close readings of the free-response data carried out by ET identified responses to the dialogical form of the two texts and responses to the experimental setup as possible mechanisms mediating the substantial positive effects of time but not text.

#### Responses to dialogue and question/answer structures in the texts

“I feel very grateful to have taken part and I think that the concept of the book is great although for me personally it did not work well. It is amazing and intense to get the insights straight in direct speech. […]” (HA participant)

We suggested in the Results section that participants’ responses to the dialogical and question-and-answer form may have contributed to the positive main effects observed. These responses in the HA group reflected, to varying degrees, all four of the potential effects of this rhetorical choice listed in the Introduction:

- The alternating expression of and challenges to the ED perspective (e.g. “[…] In comparison to other memoirs I have read the ‘B’ voice acted as a kind of ‘leveller’ or reminder of rationality in the conversation. […]”)
- The enhancement of insight through dialogical interplay (especially as a three-way interaction involving the reader, e.g. “[…] as I read the questions [posed by “B” to “A”] I tried to answer them myself. As I am now in the middle of recovery, whereas when I first started reading section one I was deep in my anorexia, I could feel the way my anorexic self would have answered the questions, and compare it to how I would approach them now. […]”)
- The distinct roles of dialogical reflection versus behavioural change (e.g. “[…] i hadn’t realised until right at the end that A and B (and C) were different voices in her head. I feel like that was a good way to explore how stuck in your head you can be in the depths of your eating disorder. […] // […] Emelia was now whole again, it was one monologue rather than multiple voices, and her life was so much bigger than food. But her life wasn’t perfect - she still had problems, she still had issues with her body image some times and comparisons. But it was great that she didn’t fall back into bad habits.”)
- And the possibility of recovery despite ambivalence (e.g. “[…] The increased coming together of A and B towards the end of the section (them sharing more certainties) was very reassuring, though, in that it encapsulates the shifts of identity that seem so scary right before recovery and that are actually a lot easier to accept (and welcome) and feel more natural once recovery is taking place. […]”)

One way in which the dialogical and interrogative form may have contributed to beneficial effects for participants is in allowing for a less passive readerly interaction than a more traditionally constructed memoir. One HA participant noted at timepoint 1: “[…] The question-answer format of this section sort of ensures that it be necessarily more thought-provoking than most anorexia recovery books, and demands more active (and self-critical) engagement from the reader, which I found to be very interesting and useful in terms of imagining my recovery.” This suggests a role for empowering alternatives to the typically didactic nature of the self-help book, which can be paradoxically dis-enabling (D. Holloway, personal communication). At timepoint 2, the same participant continued: “[…] I suppose reading this section while in recovery sort of recreates the form of the text itself (the splitting of the speaker into A/B/C etc). Overall I’ve found it quite a therapeutic experience, in that it’s been a bit like performing therapy on myself.” Interestingly, this sense of enhanced agency in using the text for personal growth was experienced also by TZ participants. One made the comparison with self-help books explicit, emphasising specifically the greater autonomy invited here: “[…] I’ve appreciated reading a book that is not the typical self-help book, where you’re given a templet of things you should do, think and feel: first do A, then B, then C. If I haven’t completely misunderstood this book, it opens up to bigger, more philosophical questions (rather than more practical ones), which the reader can think about and figure out herself. And I appreciated that.”

Overall, the reports of significant cognitive demands made by both texts indicate that readers found the interpretive process challenging—in some cases off-puttingly so, in others enjoyably, but perhaps in either case as a major contributor to important forms of cognitive-emotional exploration and meta-reflection. Participants’ responses testified to the complexity of their responses, in which “positive” and “negative” cannot be neatly separated out, and in which difficult or uncomfortable experiences often form part of a broadly enriching process. This characteristic aligns with the group-reading findings alluded to earlier [33] in which liking and enjoyment did not contribute substantially to the perceived value of participation, and offers a way of accounting for readerly benefits (as indicated by the large effects on the two standardized measures) that does not depend on an anodyne concept of bibliotherapeutic change. The editing process prior to publication will preferentially target the elements of confusion in HA that can be reduced without compromising the intended ambiguities and complexities.

#### Responses to experimental setup

[Please, wait until tomorrow to carry on reading. Thank you.]

“Roger that. Thank you for these little snippets of kindness and curiosity. Cheers.” (TZ participant)

As we ask what interactions of textual features and cognitive processing may be driving the EDE-Q and ANSOCQ improvements, it is also possible that the text itself is something of a red herring. The nature of the reading and interpretive processes themselves may exert stronger effects than the linguistic content on which they centre. This would converge with the findings in Carney & Robertson [12] showing that positive effects of reading fiction on mood and wellbeing may emerge only once readers are given the opportunity to reflect on the text they have read, whether through recall or discussing texts with other readers. That is, merely *reading* a text is not enough for the benefit of the text to be actualized. In the present study, participants were invited to reflect on HA and TZ via staged questions at regular timepoints, thereby providing a context for reflection that was at once sustained and semi-social. On this view, the otherwise puzzling presence of an effect for time and no effect for textual condition becomes explicable as the result of giving readers an ideal scenario for reflective consolidation, where the fact of consolidation is more important than the details of the texts being read. This account also aligns with the “absolute sleeper effect”, in which readers continue to think and be influenced by what they have read in a book long after finishing it [35].

Such results raise the further possibility that the methods designed here to elicit free-response testimony on the reading experience and its effects and context may have substantively changed the processes under investigation. This is to some extent inevitable with any investigative procedure: Volunteering for a research study and reading a book in a specific format, within a specific timeframe, accompanied by a range of self-report tasks, cannot leave the reading experience unaltered relative to recreational reading. Reactivity effects are well documented in psychology and health contexts, with the fact of being observed or measured generally increasing positive behaviours and reducing negative ones (for an overview, see [36]), and self-tracking showing similar positive effects [37]. The effects of the qualitative aspect of the study design may be wider-ranging than expected, however. The value of responding to recurring questions throughout the reading process was mentioned by several participants, including as transferable to everyday life, while others disliked certain aspects of the question-answering procedure or suggested alternatives, and others again used the questions as prompts to what we might identify as self-coaching.

Our first recurring question, “How is your day going?”, functions similarly to the most basic of journaling prompts used by millions of individuals worldwide every day. Some researchers on expressive writing and its therapeutic benefits have suggested that exploration of self through writing is less meaningful if it does not encompass a wide temporal span taking in the moderate to distant past. In a diary-writing study, for example, Green [38, p142] reports that the group who received a long Wordsworth poem to reflect on in their writing showed evidence of a broad range of memories being elicited by the text, whereas the control group’s entries “concentrated overwhelmingly on the daily routines and habitual preoccupations of each participant”, with the strong implication that the control group’s writing was therefore less meaningful or valuable. But the search for the origins of illness may often be a misguided endeavour in the context of mental health [39] and a cognitive-behavioural perspective would suggest that the benefits to be derived from conceptual/linguistic interrogation of one’s self and life are likely to accrue predominantly by shifting the interactions between present and future rather than by taking up a new explanatory stance on the past. The present study’s findings are compatible with the idea that meaningful insights and the potential for meaningful change can arise from prompts and focal points that are not primarily past-oriented, and via textually inflected reflection that does not involve significant amounts of recall or interpretation of distant memories. Overall, the combination of open-ended prompts to reflection plus the communicative structure of respondent and recipient may have contributed to a process with qualitatively different interpretive outputs relative to reading a text with only the pre- and post-reading questionnaires.

### Limitations and future directions

We have speculated as to the possible mechanisms underlying the main effects found in this study: the significant improvement for both groups on the EDE-Q and (with an especially large effect size) the ANSOCQ. We have identified potential contributors amongst contextual factors in the experimental setup (specifically the recurring open questions) and features of textual construction (dialogue form). Insights yielded by other aspects of the free-response data—on dimensions including triggering, cognitive-emotional exploration, and identification and related phenomena—will be reported in a separate publication. More research will be needed to establish causal dynamics with confidence, particularly with respect to the question of the relative contributions of textual versus contextual factors. Resources permitting, inclusion of an assessment-only condition, in which two of the three recurring questions (excluding the reading-focused one) would be administered with no reading task, could shed further light on this question in future studies. On the question of thematic emphasis, further inquiry is called for into the distinction between ED-themed and non-ED themed narratives, which previous observational data [5] suggested is fundamental but whose significance the current experimental findings call into question.

In the present study, we used two unrelated texts as experimental and control text, cognisant of the fact that there is no such thing as either a perfectly matched text (any given pair of texts will vary on multiple dimensions) or a perfectly neutral text (every text has complex and partially unpredictable effects on multiple dimensions for different readers at different times). The texts were significantly different in length (HA was much longer), which may have created uncontrolled-for variance in responses. In previous work [26], we used one text in two versions, differentiated by the changes that the author (Franz Kafka) himself made during the writing process, and argued that this method offers a strong combination of comparability and ecological validity. This method is often not viable for texts that are not equipped with the extensive scholarly apparatus characteristic of the Western canon. In practice, other experimental/control combinations are also needed, and future investigations into health-related effects of reading will depend on generating appropriately robust but realistic paradigms.

Using a control group for reading studies can pose difficulties specifically in relation to participant expectations and demand characteristics. Here, HA participants received the type of book they were expecting, while TZ participants were not expecting to read a book about meditation and so may have reacted to it in ways that would be uncharacteristic of individuals who had chosen to read such a book—for example, reading faster, skipping bits, or reading with less interest altogether. Some TZ participants indicated that this had been the case in their reading. Specifying in the recruitment materials that meditation would be the control text subject matter might have helped reduce problems with participants finding TZ boring or dropping out altogether. (The TZ group had 63 dropouts versus 53 in the HA group, though mostly before beginning to read.) However, some level of disappointment not to be assigned to the experimental group may be inevitable.

In future studies we would advise including an initial reading checkpoint at the very start of the text, in order to generate more accurate data about total reading duration. We estimate that the mean duration here was approximately one month, but this involves rough estimates of the gap between the start of reading (as opposed to when text access was provided) and the first checkpoint being reached. There were 3 outliers who took a long time to complete the reading: one TZ participant who took around 10 months, and two HA participants who took three and four months respectively. Data collection for this study took a total of 13 months, a relatively long duration that may have reduced data consistency both at an individual level (with more potential for changes in life circumstances and events) and across the groups (especially considering that the year between summer 2021 and 2022 was a year of significant change globally in relation to the progression of the Covid-19 pandemic).

The post-reading measures were taken two weeks after reading, aiming to avoid measuring too soon and failing to capture the persistent and potentially increasing aftereffects of reading complex texts [35] and also measuring too late to capture possible short-term “triggered” effects. The timing of the post-measure meant that the full two suggested weeks of reading would be captured in the questionnaire responses. We opted not to include a longer follow-up phase in order to avoid amplifying recruitment challenges, but in future studies a light-touch follow-up after approximately three months would be advisable to test for maintenance or loss of the post-reading effects. Future studies should take into account the possibility that initial positive effects may be short-lived or conversely that short-term negative effects may be precursors to longer-term benefits, potentially via uncomfortable confrontation of difficult realities.

## Conclusion

In this study, readers read either an ED recovery memoir or a control text unrelated to EDs. The memoir was designed to reduce the likelihood of damaging responses amongst readers with a current self-reported ED, in particular by virtue of focusing equally on recovery and illness and by employing a dialogue format rather than a more conventional consistently focalized narrative form. Participants answered open-ended questions at intervals during the reading, as well as completing two clinical questionnaires, the EDE-Q and the ANSOCQ, one week before and two weeks after reading. Participants in both groups manifested significant improvement on both the EDE-Q and the ANSOCQ. No effects were found for age, duration of professional support, education, or BMI change. In the free-text responses, values for interoception, olfaction, gustatory, mouth, torso, and hand/arm were higher in responses to the ED text than to the control text, and a range of significant within-participant changes were found for sensory and action-effector dimensions at the six reading timepoints. For both groups, the free-text responses evinced linguistic similarity to the text that had been read, more so for the ED text than the control.

This experiment was designed to dictate a yes/no decision about whether to publish the ED memoir, and to guide pre-publication edits if so. The threshold for publication was a statistically defined measure of significant harm, i.e. the requirement was merely not to do significant harm rather than to do demonstrable good. In fact, benefits accrued on the two quantitative measures employed. The memoir will now be published, with edits undertaken in particular to render the dialogue format less confusing. The study sets a precedent for pre-publication ethics-testing of potential publications in sensitive domains, and encourages a more consistent separation of the writing process (which may benefit the author) and the decision of whether to publish (which may or may not harm or benefit anyone else).

In rhetorical terms, the ED memoir under investigation developed dialogical elements found in some other ED narratives, which have potential to induce a range of interpretive processes that are relevant to illness and recovery whilst reducing the risk of provoking “triggered” responses in readers. These effects may be enhanced by availability of a structured framework for processing, as provided here in the form of the regular open-ended prompts to reflection—a structure that was intended to gather qualitative data about participants’ reading experiences but may have affected these experiences more profoundly than anticipated. These findings build on previous evidence suggesting that bibliotherapeutic interventions may be enhanced by systematic interpretive supports [9] and confirms that these may not need to be elaborate or intensive. Structured interpretive engagement can be promoted by simple automated methods.

Given the broader need for reliably effective therapies for EDs, especially for AN [39], the current study was motivated in part by the possibility that narratively mediated attitude change may enhance treatment efficacy by reducing individuals’ ambivalence about illness and recovery (expressed by many of our participants) and aligning their intentions and aspirations more fully with the concept of full recovery. In this sense, we anticipated that benefits might accrue from the reading process that would not be detectable by standardized clinical severity measures. In reality, however, clinical severity was also significantly impacted by the passage of time (here taken as a plausible proxy for the reading process), if not by the specific texts themselves. This core finding implies that narrative reading should be considered both more sceptically (maybe the specific narratives matter less than we thought) and less sceptically (maybe the narrative reading process really can bring about meaningful change) than our instincts may suggest.

## Data Availability

The pre/post-test EDE-Q and ANSOCQ data, as well as the data used to support the analysis presented in the sections on close reading of the free-text responses, are available in the Oxford University Research Archive (ORA) repository, https://ora.ox.ac.uk/objects/uuid:f1a148db-5d16-4c8b-9a0b-090bf3787433, unique persistent identifier 10.5287/ora-7eomyxoy1. Since the free-text data content is sensitive, requests for full access will be considered on a case-by-case basis.
